# Biologics for Reducing Cardiovascular Risk in Psoriasis Patients

**DOI:** 10.3390/jcm12031162

**Published:** 2023-02-01

**Authors:** Hitoshi Terui, Yoshihide Asano

**Affiliations:** Department of Dermatology, Tohoku University Graduate School of Medicine, 1-1 Seiryomachi, Aoba-ku, Sendai 980-8574, Japan

**Keywords:** psoriasis, cardiovascular disease, systemic therapy, biologics

## Abstract

Psoriasis is a chronic inflammatory skin disease with a high prevalence of cardiovascular disease (CVD), obesity, dyslipidemia, hypertension, diabetes mellitus, and metabolic syndrome. Among them, CVD is the most common cause of morbidity and mortality in psoriasis patients. Since CVD is associated with considerable morbidity and mortality, primary care clinicians are increasingly committed to reducing the risk of CVD in patients with psoriasis. Biologics targeting TNF-α, IL-12/23, and IL-17 are systemic therapies that can dramatically improve the condition of psoriasis. Recent studies have reported that these inflammatory cytokine signals may promote atherosclerosis, suggesting that biologics might be effective for improving psoriasis as well as reducing the risk of CVD. Here, we reviewed cardiovascular risk in psoriasis patients, the association between psoriatic inflammation and atherosclerosis, and the efficacy of biologics for reducing the risk of cardiovascular diseases.

## 1. Introduction

Psoriasis is a chronic inflammatory skin disease characterized by erythema with scaling. It affects 2–3% of the world’s population and about 0.5% of Asians [1,2,3,4]. Innate and acquired immunity is involved in the pathogenesis of psoriasis. Moreover, psoriasis is an independent risk factor for cardiovascular events, and the cardiovascular risk is particularly high in patients with severe psoriasis [5]. The increased prevalence of cardiovascular risk factors such as obesity, dyslipidemia, hypertension, diabetes mellitus, and metabolic syndrome in patients with psoriasis is also associated with an increased risk of developing cardiovascular diseases such as myocardial infarction, angina pectoris, and stroke [6,7,8,9]. Chronic inflammation is considered a strong link between psoriasis and associated cardiovascular events [10]. Various cytokines and inflammatory cells play a central role in developing psoriatic lesions, resulting in endothelial dysfunction [11,12]. Recently, the concept of the “psoriasis march” has been proposed, in which systemic inflammation caused by psoriasis and obesity leads to insulin resistance and vascular endothelial dysfunction, which in turn promotes atherosclerosis and the development of cardiovascular disease (CVD) [13]. It is essential to note the high incidence of CVD in patients with psoriasis, since CVD is directly related to morbidity and mortality. This means that cardiovascular risk should be assessed in patients with psoriasis, and lifestyle modifications should be made to manage blood pressure, blood glucose, and lipids. In addition, strict therapeutic control is also important to control the systemic inflammation of psoriasis. The advent of biologics has dramatically changed the treatment of psoriasis. TNF-α inhibitors, IL-23 inhibitors, and IL-17 inhibitors are highly effective against psoriatic skin lesions [14,15,16,17,18,19,20,21]. Recently, clinical studies and basic research have suggested that these biologics, which target inflammatory cytokines, effectively reduce cardiovascular risk [11,22,23,24,25,26,27,28,29]. This article discusses cardiovascular risk in psoriasis, the association between psoriatic inflammation and atherosclerosis, and the cardiovascular-risk-reducing effects of biologics.

## 2. Cardiovascular Events and Modifying Factors in Psoriasis Patients

Psoriasis is a systemic chronic inflammatory disease associated with various complications such as CVD, metabolic syndrome, obesity, hypertension, dyslipidemia, and diabetes mellitus [6,7,8,9]. Psoriasis arthritis also increases the risk of CVD [30]. Previous studies have shown high rates of obesity in patients with psoriasis. In Germany, a national cross-sectional survey showed that the BMI of psoriasis patients is 28.0, which is higher compared to 25.9 in normal subjects [2]. In a Japanese study, the ratio of obesity/overweight in psoriasis patients was 39.7%, and that in control patients was 22.7%. The OR for obesity in psoriasis patients was 2.24 (95% CI 1.36–3.69) [3]. In a meta-analysis, the pooled OR for obesity among patients with psoriasis was 1.66 (95% CI 1.46–1.89) compared with those without psoriasis. The psoriasis severity was also associated with obesity. The pooled OR for obesity among patients with mild psoriasis was 1.46 (95% CI 1.17–1.82), and the pooled OR for patients with severe psoriasis was 2.23 (95% CI 1.62–3.05) [4]. Moreover, obesity could influence the therapeutic approach and clinical response to biologics. In fact, Enos CW et al. showed that obesity could reduce the efficacy of TNF-α inhibitors and IL-17 inhibitors in psoriasis patients [5]. We have recently shown that high BMI or high HbA1c were associated with secondary failure and discontinuance of infliximab [6]. These findings suggest a high prevalence of obesity in patients with psoriasis, and the effect of biologics is diminished in obese individuals with psoriasis. Adipose tissue has an important role not only in the development of systemic inflammation but also in the contribution to obesity-associated CVD risk. Adiponectin is an adipocyte-specific secretory protein present in the circulation, eliciting protective effects in the vasculature and myocardium. However, plasma levels of adiponectin in psoriasis are negatively correlated with PASI [7]. This might be one explanation for the high prevalence of CVD in psoriasis patients. In Italy, a randomized controlled clinical trial was conducted to assess the impact of a dietary intervention combined with physical exercise for weight loss on improving psoriasis in overweight or obese patients [8]. Intention-to-treat analysis showed a median PASI reduction of 48% (95% CI 33.3–58.3) in the dietary intervention arm and 25.5% (95% CI 18.2–33.3%) in the information-only arm [8]. Encouraging overweight or obese patients with psoriasis toward diet restriction and the promotion of physical exercise can help to reduce psoriasis severity, leading to an increase in the level of adiponectin and subsequently to prevention of the development of CVD.

Hypertension is more prevalent among psoriasis patients and is associated with the severity of psoriasis. A prospective cohort study revealed that psoriasis is associated with an increased risk of hypertension [9]. Moreover, psoriasis patients tend to have difficulty managing hypertension compared to non-psoriatic hypertensive patients. A case–control study showed that, compared to non-psoriatic hypertensive patients, psoriasis patients with hypertension were 5 times more likely to be on a monotherapy antihypertensive regimen (95% CI 3.60–7.05), 9.5 times more likely to be on dual antihypertensive therapy (95% CI 6.68–13.65), 16.5 times more likely to be on a triple antihypertensive regimen (95% CI 11.01–24.84), and 19.9 times more likely to be on quadruple therapy or a centrally acting agent (95% CI 10.58–37.33) in multivariable analysis after adjusting for traditional cardiac risk factors [10]. In addition, a population-based cross-sectional study showed an increased likelihood of uncontrolled hypertension among patients with more severe psoriasis, independent of other risk factors, such as BMI [11].

Increased risk for diabetes has been found in psoriasis patients [12]. Armstrong AW conducted a meta-analysis showing that psoriasis is associated with an OR of 1.59 (95% CI, 1.38–1.83) for diabetes. The pooled OR is 1.53 (95% CI, 1.16–2.04) for mild psoriasis and 1.97 (1.48–2.62) for severe psoriasis [13]. Multiple proinflammatory cytokines, such as TNF-α, IFN-γ, or IL-17, were elevated not only in skin lesions but also in circulation. These proinflammatory cytokines promote insulin resistance, which eventually induces beta-cell failure and leads to the development of diabetes [14]. Furthermore, diabetic patients with psoriasis develop microvascular events more than patients without psoriasis. The distinct mechanistic association between psoriasis and diabetes is not completely understood. However, it seems almost evident that managing both diseases and controlling symptoms would decrease the incidence of CVD.

Psoriasis relates to dyslipidemia, which is also a factor of risk for CVD. In a systemic review, 20 out of 25 studies, including 265,512 psoriasis patients, reported that psoriasis was significantly associated with dyslipidemia. The OR for dyslipidemia ranged from 1.04 to 5.55 in 238,385 psoriasis patients [15]. The serum levels of triglyceride, cholesterol, and LDL were significantly higher in psoriasis patients, but not HDL levels [16]. Furthermore, TNF-α and other proinflammatory cytokines promote dyslipidemia by increasing the levels of LDL-C and oxLDL-C, decreasing the quality of lipoprotein, and reducing the level of HDL-C [17,18]. Using nuclear magnetic resonance spectroscopy, the lipid profile in psoriasis patients is similar to that observed in diabetes patients [19]. These lipid abnormalities in psoriasis patients drive systemic inflammation and promote insulin resistance, finally leading to the development of CVD.

Furthermore, many studies have found psoriasis to be an independent risk factor for atherosclerosis, myocardial infarction, stroke, and diabetes [5,31,32,33,34,35,36,37,38,39,40,41,42,43,44,45,46,47,48,49,50,51]. In particular, CVDs such as atherosclerosis, myocardial infarction (MI), and stroke are among the most critical complications because they are fatal. To investigate the causes of death in psoriasis, a large cohort study was conducted in the United Kingdom from 1987 to 2002. Comparing 3603 severe psoriasis patients with 14,330 healthy controls, the study found that life expectancy was approximately six years shorter in patients with severe psoriasis. Cardiovascular events were the most common cause of death [52]. Table 1 lists epidemiological studies published between 2006 and 2021 that examined the association between psoriasis and CVD [5,9,49,52,53,54,55,56,57,58,59,60,61,62,63,64,65,66,67,68,69,70,71,72,73,74,75,76].

**Figure 1:**
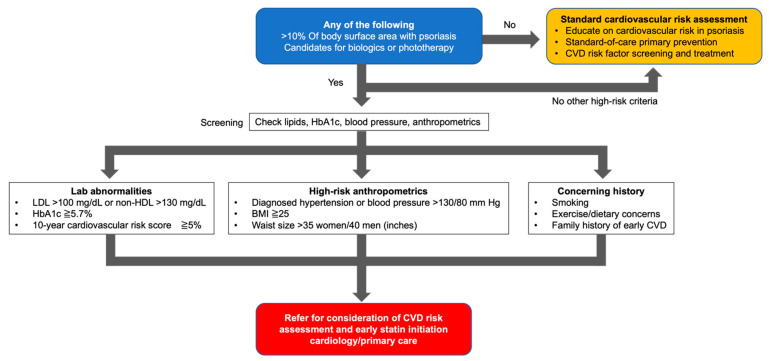
Suggested role of primary clinicians in primary cardiovascular prevention in psoriasis patients. Adapted from Garshick, M.S. and Berger, J.S. JAMA dermatology [84]. BSA: body surface area, CVD: cardiovascular disease, BMI: body mass index.

CVD included MI, coronary artery disease, angina, atherosclerosis, peripheral vascular disease, stroke, ischemic heart disease, cerebrovascular disease, CVD mortality, and coronary heart disease. Most studies found that the presence of psoriasis increased the risk of CVD, although some studies found no association between psoriasis and CVD risk. The risk of MI and stroke in mild psoriasis and severe psoriasis requiring systemic therapy was generally increased in severe psoriasis. As described above, many epidemiologic studies have been conducted. A systematic review including these epidemiological studies revealed that psoriasis increases the risk of CVDs such as MI and stroke [77,78,79], and statin administration is recommended to reduce CVD risk in patients at risk for CVD [80,81]. However, in practice, only a small percentage of physicians prescribe statin administration to those who need statin treatment [81]. Therefore, Barbieri et al. investigated measures to improve CVD prevention through specialist-led care from the perspective of healthcare professionals and patients. Results of the study showed that dermatologists and psoriasis patients had a positive view of participating in a specialist-led model of care to improve CVD prevention [82]. This suggests that dermatologists need to be more proactive in communicating with psoriasis patients and engaging in statins and other treatments to reduce CVD risk in psoriasis patients. The American Academy of Dermatology and National Psoriasis Foundation recommend assessing the risk of CVD in psoriasis patients. The screening of hypertension and diabetes and the assessment of CV risk every 3–6 years are encouraged for psoriasis patients [83]. In an effort to reduce CVD risk in patients with psoriasis, Garchick and Berger et al. proposed an algorithm to be used in patients with psoriasis who have a BSA >10% or who require biologic agents or phototherapy (Figure 1) [84]. If the patient is at high risk, an appropriate CVD risk assessment should be performed, and the initiation of statin therapy should be considered. Even if the patient is not at high risk, performing a standard cardiovascular risk assessment is essential. Since dermatologists are the primary point of contact for psoriasis care, it is important for us to keep in mind that patients with severe psoriasis should be screened for a thorough CVD risk assessment.

## 3. Atherosclerosis in Psoriasis Patients

It is known that coronary artery plaques are more common in patients with psoriasis than in healthy individuals [50]. In the pathogenesis of psoriasis, inflammatory cytokines such as TNF-α, IL-23, and IL-17 form the primary pathogenesis and cause systemic inflammation. These inflammatory cytokines may cause vascular damage not only in the skin, but also in other parts of the body, and increase the risk of developing CVD via atherosclerosis. In fact, psoriasis and atherosclerosis have much in common [3,4,85,86]. It has been reported that treatment targeting IL-1β reduces atherosclerosis [24]. Therefore, it has been suggested that treatment targeting TNF-α, IL-23, and IL-17, which play a major role in shaping psoriasis pathology, may also contribute to the reduction in atherosclerosis.

**Figure 2:**
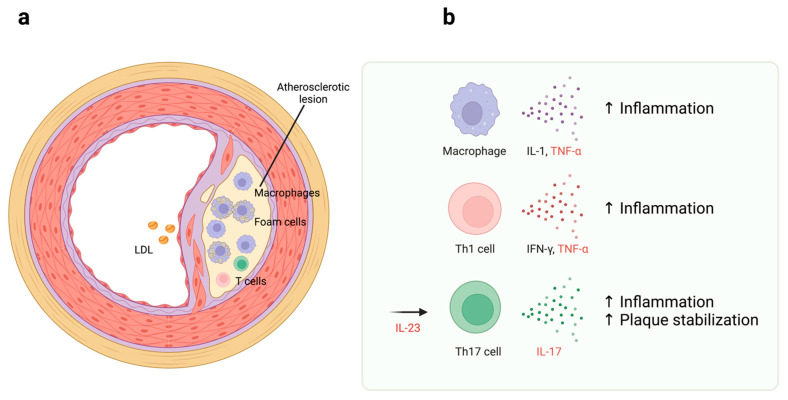
Immune cells in atherosclerosis. (**a**) LDL enters the vessel wall from blood circulation. Macrophages uptake oxidized LDL and transform it into foam cells. Macrophages, as well as T cells, are involved in advancing atherosclerosis. (**b**) Macrophage and Th1 cells produce IL-1, TNF-α, and IFN-γ for promoting inflammation. Th17 cells produce IL-17, which has both a pro-atherosclerotic function promoting inflammation and an anti-atherosclerotic function stabilizing atherosclerotic plaque. TNF-α, IL-23, and IL-17 (in red text) are current molecular targets of biologics used in psoriasis patients. Accumulating evidence suggests that inhibiting the function of these molecules might reduce the risk of CVD. The illustrations were created with BioRender, https://biorender.com/, accessed on 15 January 2023.

Risk factors associated with atherosclerosis, such as hypertension and diabetes mellitus, increase vascular endothelial damage. High levels of LDL cholesterol in the blood gradually lead to excessive accumulation of LDL cholesterol in the intima of blood vessels. The accumulated LDL cholesterol is oxidized by reactive oxygen species and converted to oxidized LDL. Macrophages take up the oxidized LDL via scavenger receptors and become foam cells. Over time, the foam cells promote atherosclerosis by promoting atheroma plaque formation [87]. Macrophages in the plaque release inflammatory cytokines such as IL-1 and TNF-α. These inflammatory cytokines are thought to promote further atherosclerosis [88,89] (Figure 2).

Various studies have shown that acquired immunity also plays a vital role in atherosclerosis. It has been found that CD4+ T cells are present in atheroma plaques [90]. CD4+ T cells also play a significant role in the pathogenesis of psoriasis, especially Th1 cells and Th17 cells. Th1 cells differentiate and produce IFN-γ under the action of IL-12. IFN-γ is also involved in the production of proinflammatory cytokines, the upregulation of gene expression of adhesion molecules, the production of psoriasis-related cytokines, and the activation of macrophages and vascular endothelial cells, leading to the development of atherosclerotic lesions [91,92].

IL-17 is a cytokine produced by CD4+ T cells. Th17 cells are activated mainly through cytokine signaling from dendritic cells [3]. The IL-17 receptor is expressed on epidermal cells in the skin, and IL-17 activates epidermal cells to cooperate with inflammatory cells to form the psoriasis pathology. The IL-17 receptor is known to be expressed on vascular endothelial cells, and its action on vascular endothelial cells is believed to promote the production of inflammatory cytokines such as granulocyte colony stimulating factor (G-CSF) and IL-6, promoting atherosclerosis [93]. On the other hand, other reports indicate that IL-17 does not promote atherosclerosis. In addition, IL-17 stabilizes plaque and may have an anti-atherogenic effect.

## 4. The Effect of Psoriasis Treatments on Cardiovascular Risk

The main strategy to reduce the risk of CVD has been the prevention of atherosclerosis through strict lipid control. In fact, the risk of CVD can be reduced by statin administration [80,94]. More recently, it has been found that statin administration reduces vascular endothelial inflammatory markers [95]. Thus, it is clear that lipid control is important in reducing CVD risk. In addition, the recent success of the CANTOS (Canakinumab Anti-inflammatory Thrombosis Outcomes Study) trial has established evidence that the suppression of inflammatory cytokines reduces CVD risk. In this study, the anti-inflammatory effects of IL-1β inhibitor canakinumab administration were compared between three different doses and placebo in 10,061 patients with a history of myocardial infarction and high-sensitivity C-reactive protein levels. The results validated that anti-inflammatory therapy with canakinumab reduced the recurrence of CVD events [24]. Moreover, a recent study showed that biologics could reduce coronary inflammation assessed as perivascular fat attenuation index [96]. Furthermore, biologics can decrease intima–media thickness (IMT), an indicator of subclinical atherosclerotic plaque development, by reducing inflammation [80,86,87,88,89,90,91,92,93,94,95,96,97,98,99,100]. In fact, cardiovascular events were reduced in psoriasis patients with biological treatment (HR 0.58 (0.30–1.10)) in a Danish nationwide cohort study [101]. Therefore, anti-inflammatory therapy targeting TNF-α, IL-23, and IL-17, the main pathological factors in psoriasis, can potentially contribute to the reduction in CVD risk and psoriatic skin rash.

Weight gain and higher BMI have been reported with TNF-α inhibitors; weight loss should be requested at the same time when using TNF-α inhibitors [102,103]. TNF-α inhibitors have been reported to be effective in treating insulin resistance in psoriasis patients [104], but no such reports have been reported for other biologic agents. Meta-analysis suggests that TNF-α inhibitor treatment reduces CVD risk [105,106]. The risk of myocardial infarction was also reduced with TNF-α inhibitors [107]. While these clinical studies suggest that TNF-α inhibitors may contribute to CVD risk reduction, some studies have shown the opposite. In summary, there is currently disagreement as to whether TNF-α inhibitors can reduce the risk of CVD, and further accumulation of evidence is needed.

IL-23 is a cytokine composed of two subunits, IL-12p40 and IL-23p19. IL-23 induces Th17 cell differentiation. The IL-23/IL-17 axis is central to psoriasis pathogenesis, and blocking IL-23 is highly effective for psoriasis lesions. Some studies showed the proatherogenic role in IL-23. IL-23 is highly expressed in atherosclerotic lesions [108], serum levels of IL-23 are elevated in patients with carotid atherosclerosis compared to healthy controls, and IL-23 and IL-23R mRNA levels are significantly elevated in carotid plaques. It has also been found that patients with elevated serum IL-23 levels have higher mortality [25]. Granulocyte macrophage colony-stimulating factor (GM-CSF) promotes plaque progression, which is mediated by IL-23, and increases apoptosis susceptibility in macrophages by promoting proteasomal degradation of the IL-23-mediated apoptosis susceptibility in macrophages by promoting proteasomal degradation of the cell survival protein B-cell lymphoma 2 (Bcl-2) and by increasing oxidative stress [26]. Information on IL-23 inhibitors’ CVD risk reduction remains at the research level, with some studies suggesting that IL-23 is protective against atherosclerosis by acting to maintain the intestinal barrier and homeostasis of the intestinal microbiota [109]. On the other hand, some studies using LDL receptor-deficient mice have shown that IL-23 does not affect atherosclerosis [110].

IL-17A, IL-17B, IL-17C, IL-17D, IL-17E, and IL-17F are known as IL-17 family members [111]. Secukinumab, ixekizumab, bimekizumab, and brodalumab as IL-17 inhibitors have been used to treat psoriasis [14,16,18,19]. IL-17A and IL-17F are mainly associated with psoriasis, forming homodimers of each or heterodimers of both subunits [111]. Secukinumab and ixekizumab target IL-17A, and bimekizumab targets both IL-17A and IL-17F. On the other hand, brodalumab targets the IL-17 receptor, IL-17RA. These IL-17 inhibitors have also shown efficacy against psoriatic skin lesions in direct comparative studies with etanercept and ustekinumab [18,112,113]. The effect of IL-17A on vascular dysfunction has been examined in a mouse model; administration of angiotensin II increases IL-17 protein from T cells and aortic media. Mice showed improvement in blood pressure elevation and vascular dysfunction associated with administering angiotensin II [29]. Another laboratory has shown that blocking IL-17A reduces peripheral oxidative stress levels, proinflammatory cytokines, and vascular inflammation [23]. Furthermore, there is a report that IL-17A expression is high in atherosclerotic plaques from patients with ischemic symptoms [108]. These results show that IL-17A increases plaque instability and causes atherosclerosis. On the other hand, the results from other researchers demonstrated that IL-17A has a protective role in atherosclerosis. IL-17A levels are not involved in atherosclerotic plaque formation because there is no correlation between serum IL-17A levels and carotid intima–media thickness [27]. Thus, reports on IL-17A and atherosclerotic plaque formation have yielded different views. In 2019, the benefit of secukinumab, an IL-17A antagonist, for cardiovascular markers was investigated in the CARIMA (Evaluation of Cardiovascular Risk Markers in Psoriasis Patients Treated with Secukinumab) study. In this study, patients with moderate to severe plaque psoriasis without clinical CV disease were treated with secukinumab, and endothelial function was measured by flow-mediated dilation (FMD) as the primary endpoint. Baseline FMD was predominantly lower in psoriatic patients compared to healthy volunteers; secukinumab did not make a difference at 12 weeks, but there was an increase in FMD in psoriatic patients receiving secukinumab at 52 weeks [11]. Furthermore, another recent study, a prospective observational study for coronary artery plaque characteristics in psoriasis patients with biologics, showed IL-17 inhibitors reduced non-calcified plaque burden in psoriasis patients, suggesting the crucial role of IL-17 in atherosclerotic pathways [22]. Although future extensive clinical studies are needed, this is an important study that suggests a reduction in CVD risk by suppressing IL-17A.

## 5. Conclusions

Psoriasis is not only a cutaneous but also a systemic inflammatory disease, a group of diseases that carries a very high cardiovascular risk. Therefore, it is necessary to break away from treating psoriasis as a skin-only target and treat it as a systemic disease to avoid highly lethal cardiovascular disorders. We need to focus on obesity, dyslipidemia, diabetes, hypertension, and metabolic syndrome, which are frequently associated with psoriasis, and provide integrated treatment in close collaboration with other medical departments. Inflammatory cytokines, mainly TNF-α, IL-17, and IL-23, which occur in the pathogenesis of psoriasis, are thought to increase the risk of CVD, leading not only to psoriasis but also to vascular dysfunction. Therefore, it is suggested that biologics targeting these molecules may have a positive impact not only on the pathogenesis of psoriasis but also on CVD risk.

## Figures and Tables

**Table 1:** Summary of studies investigating the relationship between psoriasis and cardiovascular diseases. Hazard ratios (HRs) or odds ratios (ORs) with 95% confidence intervals.

Authors	Patients	Controls	Cardiovascular Diseases	Relative Risk	Publication	Country
Gelfand et al. [5]	Severe psoriasis: 3837, Mild psoriasis: 127,139	556,995	MI	30-year-old: Severe psoriasis: HR 3.10 (1.98–4.86), Mild psoriasis: HR 1.29 (1.14–1.46)	2006	United Kingdom
				60-year-old: Severe psoriasis: HR 1.36 (1.13–1.64), Mild psoriasis: HR 1.08 (1.03–1.13)		
Sommer et al. [9]	581	1044	Coronary artery disease	OR 1.77 (1.07–2.93)	2007	Germany
Kaye et al. [76]	44,164	219,784	MI	HR 1.21 (1.10–1.32)	2008	United Kingdom
			Angina	HR 1.20 (1.12–1.29)		
			Atherosclerosis	HR 1.28 (1.10–1.48)		
			Peripheral vascular disease	HR 1.29 (1.13–1.47)		
			Stroke	HR 1.12 (1.00–1.25)		
Brauchli et al. [73]	449	1796	MI	OR 1.14 (95% CI 0.93–1.41)	2009	United Kingdom
Xiao et al. [75]	3092	1521	MI	OR 2.11 (95% CI 1.45–3.04)	2009	China
Chiang et al. [66]	2783	13,910	Stroke	HR 1.25 (1.04–1.51)	2009	Taiwan
Gelfand et al. [49]	Severe psoriasis: 3603	14,330	Stroke	Severe psoriasis: HR 1.43 (1.1–1.9)	2009	United Kingdom
	Mild psoriasis: 129,143	496,666	Stroke	Mild psoriasis: HR 1.06 (1.0–1.1)		
Prodanovich et al. [74]	3236	2500	Ischemic heart disease	OR 1.78 (1.51–2.11)	2009	United States
			Cerebrovascular	OR 1.70 (1.33–2.17)		
			Peripheral vascular disease	OR 1.98 (1.32–2.82)		
			Mortality	OR 1.86 (1.56–2.21)		
Abuabara et al. [52]	Severe Psoriasis: 3603	14,330	CVD	HR 1.57 (1.26–1.96)	2010	United Kingdom
Wakkee et al. [72]	15,820	27,577	Ischemic heart disease	HR 1.10 (95% CI 0.99–1.23)	2010	Netherlands
Schmitt et al. [71]	3147	3147	MI	OR 1.14 (95% CI 0.81–1.62)	2010	Germany
			Stroke	OR 0.97 (95% CI 0.61–1.54)		
Mehta et al. [70]	Severe psoriasis: 3603	14,330	CVD mortality	HR 1.57 (1.26–1.96)	2010	United Kingdom
Lin et al. [68]	4752	23,760	MI	HR 2.10 (95% CI (1.27–3.43)	2011	Taiwan
Yang et al. [69]	1685	5055	Congestive heart failure	OR 1.63 (1.22–2.19)	2011	Taiwan
			Ischemic heart disease	OR 1.51 (1.02–2.43)		
Li et al. [67]	2463	96,008	Nonfatal CVD	HR 1.55 (1.04–2.31)	2012	United States
			Nonfatal MI	HR 1.70 1.01–2.84)		
			Nonfatal stroke	HR 1.45 (0.80–2.65)		
Yeung et al. [65]	9035	90,350	MI	OR 1.34 (95% CI 1.07–1.69)	2013	United Kingdom
			Peripheral vascular disease	OR 1.38 (95% CI 1.07–1.77)		
Dowlatshahi et al. [63]	262	8009	CVD	HR 0.73 (0.50–1.06)	2013	Netherlands
Levesque et al. [64]	31,421	31,421	MI	HR 1.17 (1.04–1.31)	2013	Canada
Dregan et al. [62]	Severe psoriasis: 5648, Mild psoriasis: 85,232	373,851	Stroke	Severe psoriasis: HR 0.93 (0.64–1.36), Mild psoriasis: HR 1.08 (0.98–1.18)	2014	United Kingdom
			Coronary heart disease	Severe psoriasis: HR 1.29 (1.01–1.64), Mild psoriasis: HR 1.03 (0.97–1.11)		
Koch et al. [59]	199	3986	MI	OR 2.26 (95% CI 1.08–1.14)	2015	Germany
Mahiques-Santos et al. [58]	9181	21,925	Coronary artery disease	OR 1.214 (1.053–1.399)	2015	Spain
Ogdie et al. [60]	Psoriasis: 138,424, PsA: 8706	81,573	Major adverse cardiovascular events	No DMARD: Psoriasis: HR 1.08 (1.02–1.15), PsA: HR 1.24 (1.03–1.49) DMARD: Psoriasis: HR 1.42 (1.17–1.73), PsA: HR 1.17 (0.95–1.46)	2015	United Kingdom
Wu et al. [61]	Severe psoriasis: 3841	19,205	MI	HR 1.28 (1.02–1.60)	2015	United States
	Mild psoriasis: 10,173	50,865		HR 1.31 (1.14–1.51)		
Lai et al. [57]	520	19,065	MI	OR 2.24 (1.27–3.95)	2016	United States
			Ischemic heart disease	OR 1.90 (1.18–3.05)		
			Stroke	OR 1.01 (0.48–2.16)		
Egeberg et al. [56]	Severe psoriasis: 11,957, Mild psoriasis: 49,646	4,300,085	MI	Severe psoriasis: HR 1.21 (1.07–1.37), Mild psoriasis: HR 1.02 (0.96–1.09)	2017	Denmark
Jung et al. [55]	5788	1,727,832	Atherosclerotic CVD	HR 1.18 (95% CI 1.09–1.27)	2019	Korea
Shiba et al. [54]	1197	111,868	MI	OR 1.87 (95% CI 1.26–2.68)	2019	Japan
Tinggaard et al. [53]	Psoriasis: 1356, PsA: 370	44,296	Coronary artery disease	Psoriasis: OR 1.14 (0.98–1.33), PsA OR 0.98 (0.73–1.32)	2021	Denmark
			Cardiovascular events and all-cause mortality	Psoriasis: OR 1.14 (0.92–1.41), PsA OR 1.25 (0.80–1.94)		

MI: myocardial infarction, CVD: cardiovascular disease, PsA: psoriatic arthritis.

## Data Availability

Not applicable.

## References

[1] Parisi R., Symmons D.P.M., Griffiths C.E., Ashcroft D.M. (2013). Global Epidemiology of Psoriasis: A Systematic Review of Incidence and Prevalence. J. Investig. Dermatol..

[2] Perera G.K., Di Meglio P., Nestle F.O. (2012). Psoriasis. Annu. Rev. Pathol. Mech. Dis..

[3] Nestle F.O., Kaplan D.H., Barker J. (2009). Psoriasis. N. Engl. J. Med..

[4] Lebwohl M. (2003). Psoriasis. Lancet.

[5] Gelfand J.M., Neimann A.L., Shin D.B., Wang X., Margolis D.J., Troxel A.B. (2006). Risk of Myocardial Infarction in Patients with Psoriasis. JAMA.

[6] Teklu M., Zhou W., Kapoor P., Patel N., Dey A.K., Sorokin A.V., Manyak G.A., Teague H.L., Erb-Alvarez J.A., Sajja A. (2021). Metabolic syndrome and its factors are associated with noncalcified coronary burden in psoriasis: An observational cohort study. J. Am. Acad. Dermatol..

[7] Fernández-Armenteros J., Gómez-Arbonés X., Buti-Soler M., Betriu-Bars A., Sanmartin-Novell V., Ortega-Bravo M., Martínez-Alonso M., Gari E., Portero-Otín M., Santamaria-Babi L. (2019). Psoriasis, metabolic syndrome and cardiovascular risk factors. A population-based study. J. Eur. Acad. Dermatol. Venereol..

[8] Khanna N., Kothiwala S., Tandon N., Naik N., Sharma V., Sharma S.K., Sreenivas V. (2016). Prevalence of metabolic syndrome and cardiovascular changes in patients with chronic plaque psoriasis and their correlation with disease severity: A hospital-based cross-sectional study. Indian J. Dermatol. Venereol. Leprol..

[9] Sommer D.M., Jenisch S., Suchan M., Christophers E., Weichenthal M. (2007). Increased prevalence of the metabolic syndrome in patients with moderate to severe psoriasis. Arch. Dermatol. Res..

[10] Boehncke W.-H. (2018). Systemic Inflammation and Cardiovascular Comorbidity in Psoriasis Patients: Causes and Consequences. Front. Immunol..

[11] von Stebut E., Reich K., Thaçi D., Koenig W., Pinter A., Körber A., Rassaf T., Waisman A., Mani V., Yates D. (2019). Impact of Secukinumab on Endothelial Dysfunction and Other Cardiovascular Disease Parameters in Psoriasis Patients over 52 Weeks. J. Investig. Dermatol..

[12] Lynch M., Ahern T., Sweeney C.M., Malara A., Tobin A.M., O’Shea D., Kirby B. (2017). Adipokines, psoriasis, systemic inflammation, and endothelial dysfunction. Int. J. Dermatol..

[13] Boehncke W.-H., Boehncke S., Tobin A.-M., Kirby B. (2011). The ‘psoriatic march’: A concept of how severe psoriasis may drive cardiovascular comorbidity. Exp. Dermatol..

[14] Reich K., Warren R.B., Lebwohl M., Gooderham M., Strober B., Langley R.G., Paul C., De Cuyper D., Vanvoorden V., Madden C. (2021). Bimekizumab versus Secukinumab in Plaque Psoriasis. N. Engl. J. Med..

[15] Papp K.A., Blauvelt A., Bukhalo M., Gooderham M., Krueger J.G., Lacour J.-P., Menter A., Philipp S., Sofen H., Tyring S. (2017). Risankizumab versus Ustekinumab for Moderate-to-Severe Plaque Psoriasis. N. Engl. J. Med..

[16] Gordon K.B., Blauvelt A., Papp K.A., Langley R.G., Luger T., Ohtsuki M., Reich K., Amato D., Ball S.G., Braun D.K. (2016). Phase 3 Trials of Ixekizumab in Moderate-to-Severe Plaque Psoriasis. N. Engl. J. Med..

[17] Gordon K.B., Duffin K.C., Bissonnette R., Prinz J.C., Wasfi Y., Li S., Shen Y.-K., Szapary P., Randazzo B., Reich K. (2015). A Phase 2 Trial of Guselkumab versus Adalimumab for Plaque Psoriasis. N. Engl. J. Med..

[18] Langley R.G., Elewski B.E., Lebwohl M., Reich K., Griffiths C.E., Papp K., Puig L., Nakagawa H., Spelman L., Sigurgeirsson B. (2014). Secukinumab in Plaque Psoriasis—Results of Two Phase 3 Trials. N. Engl. J. Med..

[19] Papp K.A., Leonardi C., Menter A., Ortonne J.-P., Krueger J.G., Kricorian G., Aras G., Li J., Russell C.B., Thompson E.H. (2012). Brodalumab, an Anti–Interleukin-17–Receptor Antibody for Psoriasis. N. Engl. J. Med..

[20] Gordon K.B., Langley R.G., Leonardi C., Toth D., Menter M.A., Kang S., Heffernan M., Miller B., Hamlin R., Lim L. (2006). Clinical response to adalimumab treatment in patients with moderate to severe psoriasis: Double-blind, randomized controlled trial and open-label extension study. J. Am. Acad. Dermatol..

[21] Antoni C.E., Kavanaugh A., Kirkham B., Tutuncu Z., Burmester G.R., Schneider U., Furst D.E., Molitor J., Keystone E., Gladman D. (2005). Sustained benefits of infliximab therapy for dermatologic and articular manifestations of psoriatic arthritis: Results from the infliximab multinational psoriatic arthritis controlled trial (IMPACT). Arthritis Rheum..

[22] Elnabawi Y.A., Dey A.K., Goyal A., Groenendyk J.W., Chung J.H., Belur A.D., Rodante J., Harrington C.L., Teague H.L., Baumer Y. (2019). Coronary artery plaque characteristics and treatment with biologic therapy in severe psoriasis: Results from a prospective observational study. Cardiovasc. Res..

[23] Schüler R., Brand A., Klebow S., Wild J., Veras F.P., Ullmann E., Roohani S., Kolbinger F., Kossmann S., Wohn C. (2019). Antagonization of IL-17A Attenuates Skin Inflammation and Vascular Dysfunction in Mouse Models of Psoriasis. J. Investig. Dermatol..

[24] Ridker P.M., Everett B.M., Thuren T., MacFadyen J.G., Chang W.H., Ballantyne C., Fonseca F., Nicolau J., Koenig W., Anker S.D. (2017). Antiinflammatory Therapy with Canakinumab for Atherosclerotic Disease. N. Engl. J. Med..

[25] Abbas A., Gregersen I., Holm S., Daissormont I., Bjerkeli V., Krohg-Sørensen K., Skagen K.R., Dahl T.B., Russell D., Almås T. (2015). Interleukin 23 levels are increased in carotid atherosclerosis: Possible role for the interleukin 23/interleukin 17 axis. Stroke.

[26] Subramanian M., Thorp E., Tabas I. (2015). Identification of a non-growth factor role for GM-CSF in advanced atherosclerosis: Promotion of macrophage apoptosis and plaque necrosis through IL-23 signaling. Circ. Res..

[27] Madhur M.S., Funt S.A., Li L., Vinh A., Chen W., Lob H.E., Iwakura Y., Blinder Y., Rahman A., Quyyumi A.A. (2011). Role of Interleukin 17 in Inflammation, Atherosclerosis, and Vascular Function in Apolipoprotein E–Deficient Mice. Arter. Thromb. Vasc. Biol..

[28] Ryan C., Leonardi C.L., Krueger J.G., Kimball A.B., Strober B.E., Gordon K.B., Langley R.G., De Lemos J.A., Daoud Y., Blankenship D. (2011). Association between biologic therapies for chronic plaque psoriasis and cardiovascular events: A meta-analysis of randomized controlled trials. JAMA.

[29] Madhur M.S., Lob H.E., McCann L.A., Iwakura Y., Blinder Y., Guzik T.J., Harrison D.G. (2010). Interleukin 17 Promotes Angiotensin II–Induced Hypertension and Vascular Dysfunction. Hypertension.

[30] Husted J.A., Thavaneswaran A., Chandran V., Eder L., Rosen C.F., Cook R.J., Gladman D.D. (2011). Cardiovascular and other comorbidities in patients with psoriatic arthritis: A comparison with patients with psoriasis. Arthritis Care Res..

[31] Lu Y., Chen H., Nikamo P., Low H.Q., Helms C., Seielstad M., Liu J., Bowcock A.M., Stahle M., Liao W. (2013). Association of cardiovascular and metabolic disease genes with psoriasis. J. Investig. Dermatol..

[32] Jacobi A., Langenbruch A., Purwins S., Augustin M., Radtke M.A. (2015). Prevalence of obesity in patients with psoriasis: Results of the national study PsoHealth3. Dermatology.

[33] Takahashi H., Takahashi I., Honma M., Ishida-Yamamoto A., Iizuka H. (2010). Prevalence of metabolic syndrome in Japanese psoriasis patients. J. Dermatol. Sci..

[34] Armstrong A., Harskamp C., Armstrong E. (2012). The association between psoriasis and obesity: A systematic review and meta-analysis of observational studies. Nutr. Diabetes.

[35] Enos C.W., Ramos V.L., McLean R.R., Lin T.-C., Foster N., Dube B., Van Voorhees A.S. (2022). Comorbid obesity and history of diabetes are independently associated with poorer treatment response to biologics at 6 months: A prospective analysis in Corrona Psoriasis Registry. J. Am. Acad. Dermatol..

[36] Terui H., Asano M., Shimada-Omori R., Tsuchiyama K., Takahashi T., Nasu-Tamabuchi M., Hagiwara-Takita A., Kusakari Y., Ohtani T., Aiba S. (2021). Body mass index, HbA1c and serum C–reactive protein are predictors of secondary failure in infliximab continuance for Japanese psoriasis patients: A hospital-based retrospective case—Control study. J. Dermatol..

[37] Takahashi H., Tsuji H., Takahashi I., Hashimoto Y., Ishida–Yamamoto A., Iizuka H. (2008). Plasma adiponectin and leptin levels in Japanese patients with psoriasis. Br. J. Dermatol..

[38] Naldi L., Conti A., Cazzaniga S., Patrizi A., Pazzaglia M., Lanzoni A., Veneziano L., Pellacani G., Group P.E.R.S. (2014). Diet and physical exercise in psoriasis: A randomized controlled trial. Br. J. Dermatol..

[39] Qureshi A.A., Choi H.K., Setty A.R., Curhan G.C. (2009). Psoriasis and the risk of diabetes and hypertension: A prospective study of US female nurses. Arch. Dermatol..

[40] Armstrong A.W., Lin S.W., Chambers C.J., Sockolov M.E., Chin D.L. (2011). Psoriasis and hypertension severity: Results from a case-control study. PLoS ONE.

[41] Takeshita J., Wang S., Shin D.B., Mehta N.N., Kimmel S.E., Margolis D.J., Troxel A.B., Gelfand J.M. (2015). Effect of psoriasis severity on hypertension control: A population-based study in the United Kingdom. JAMA Dermatol..

[42] Holm J.G., Thomsen S.F. (2019). Type 2 diabetes and psoriasis: Links and risks. Psoriasis Targets Ther..

[43] Armstrong A.W., Harskamp C.T., Armstrong E.J. (2013). Psoriasis and the risk of diabetes mellitus: A systematic review and meta-analysis. JAMA Dermatol..

[44] Fitzgerald R., Sadlier M., Connolly M., Tobin A.M. (2014). Psoriasis and insulin resistance: A review. J. Diabetes Res. Clin. Metab..

[45] Ma C., Harskamp C., Armstrong E., Armstrong A. (2013). The association between psoriasis and dyslipidaemia: A systematic review. Br. J. Dermatol..

[46] Akhyani M., Ehsani A., Robati R., Robati A. (2007). The lipid profile in psoriasis: A controlled study. J. Eur. Acad. Dermatol. Venereol..

[47] Shih C.-M., Chen C.-C., Chu C.-K., Wang K.-H., Huang C.-Y., Lee A.-W. (2020). The roles of lipoprotein in psoriasis. Int. J. Mol. Sci..

[48] Zhang H.H., Halbleib M., Ahmad F., Manganiello V.C., Greenberg A.S. (2002). Tumor necrosis factor-α stimulates lipolysis in differentiated human adipocytes through activation of extracellular signal-related kinase and elevation of intracellular cAMP. Diabetes.

[49] Gelfand J.M., Dommasch E.D., Shin D.B., Azfar R.S., Kurd S.K., Wang X., Troxel A.B. (2009). The Risk of Stroke in Patients with Psoriasis. J. Investig. Dermatol..

[50] Ludwig R., Herzog C., Rostock A., Ochsendorf F., Zollner T., Thaci D., Kaufmann R., Vogl T., Boehncke W.-H. (2007). Psoriasis: A possible risk factor for development of coronary artery calcification. Br. J. Dermatol..

[51] Neimann A.L., Shin D.B., Wang X., Margolis D.J., Troxel A.B., Gelfand J.M. (2006). Prevalence of cardiovascular risk factors in patients with psoriasis. J. Am. Acad. Dermatol..

[52] Abuabara K., Azfar R., Shin D., Neimann A., Troxel A., Gelfand J. (2010). Cause-specific mortality in patients with severe psoriasis: A population-based cohort study in the U.K. Br. J. Dermatol..

[53] Tinggaard A.B., Hjuler K.F., Andersen I.T., Winther S., Iversen L., Bøttcher M. (2021). Prevalence and severity of coronary artery disease linked to prognosis in psoriasis and psoriatic arthritis patients: A multi-centre cohort study. J. Intern. Med..

[54] Shiba M., Kato T., Izumi T., Miyamoto S., Nakane E., Haruna T., Inoko M. (2019). Risk of myocardial infarction in patients with psoriasis: A cross-sectional patient-population study in a Japanese hospital. J. Cardiol..

[55] Jung K.J., Kim T., Lee J.W., Lee M., Oh J., Lee S.-E., Chang H.-J., Jee S.H., Lee M. (2019). Increased risk of atherosclerotic cardiovascular disease among patients with psoriasis in Korea: A 15-year nationwide population-based cohort study. J. Dermatol..

[56] Egeberg A., Thyssen J., Jensen P., Gislason G., Skov L. (2017). Risk of Myocardial Infarction in Patients with Psoriasis and Psoriatic Arthritis: A Nationwide Cohort Study. Acta Derm.-Venereol..

[57] Lai Y.C., Yew Y.W. (2016). Psoriasis as an independent risk factor for cardiovascular disease: An epidemiologic analysis using a national database. J. Cutan. Med. Surg..

[58] Mahiques-Santos L., Soriano-Navarro C., Perez-Pastor G., Tomas-Cabedo G., Pitarch-Bort G., Valcuende-Cavero F. (2015). Psoriasis and ischemic coronary artery disease. Actas Dermo Sifiliográficas.

[59] Koch M., Baurecht H., Ried J.S., Rodriguez E., Schlesinger S., Volks N., Gieger C., Rückert I.-M., Heinrich L., Willenborg C. (2015). Psoriasis and Cardiometabolic Traits: Modest Association but Distinct Genetic Architectures. J. Investig. Dermatol..

[60] Ogdie A., Yu Y., Haynes K., Löve J., Maliha S., Jiang Y., Troxel A., Hennessy S., Kimmel S.E., Margolis D.J. (2015). Risk of major cardiovascular events in patients with psoriatic arthritis, psoriasis and rheumatoid arthritis: A population-based cohort study. Ann. Rheum. Dis..

[61] Wu J.J., Choi Y.M., Bebchuk J.D. (2015). Risk of myocardial infarction in psoriasis patients: A retrospective cohort study. J. Dermatol. Treat..

[62] Dregan A., Charlton J., Chowienczyk P., Gulliford M.C. (2014). Chronic Inflammatory Disorders and Risk of Type 2 Diabetes Mellitus, Coronary Heart Disease, and Stroke: A population-based cohort study. Circulation.

[63] Dowlatshahi E.A., Kavousi M., Nijsten T., Ikram M.A., Hofman A., Franco O.H., Wakkee M. (2013). Psoriasis Is Not Associated with Atherosclerosis and Incident Cardiovascular Events: The Rotterdam Study. J. Investig. Dermatol..

[64] Levesque A., Lachaine J., Bissonnette R. (2013). Risk of Myocardial Infarction in Canadian Patients with Psoriasis: A Retrospective Cohort Study. J. Cutan. Med. Surg..

[65] Yeung H., Takeshita J., Mehta N.N., Kimmel S.E., Ogdie A., Margolis D.J., Shin D.B., Attor R., Troxel A.B., Gelfand J.M. (2013). Psoriasis severity and the prevalence of major medical comorbidity: A population-based study. JAMA Dermatol..

[66] Chiang C.-H., Huang C.-C., Chan W.-L., Huang P.-H., Chen Y.-C., Chen T.-J., Chung C.-M., Lin S.-J., Chen J.-W., Leu H.-B. (2012). Psoriasis and increased risk of ischemic stroke in Taiwan: A nationwide study. J. Dermatol..

[67] Li W.-Q., Han J.-L., Manson J., Rimm E., Rexrode K., Curhan G., Qureshi A. (2012). Psoriasis and risk of nonfatal cardiovascular disease in U.S. women: A cohort study. Br. J. Dermatol..

[68] Lin H.-W., Wang K.-H., Lin H.-C., Lin H.-C. (2011). Increased risk of acute myocardial infarction in patients with psoriasis: A 5-year population-based study in Taiwan. J. Am. Acad. Dermatol..

[69] Yang Y.-W., Keller J., Lin H.-C. (2011). Medical comorbidity associated with psoriasis in adults: A population-based study. Br. J. Dermatol..

[70] Mehta N.N., Azfar R.S., Shin D.B., Neimann A.L., Troxel A.B., Gelfand J.M. (2010). Patients with severe psoriasis are at increased risk of cardiovascular mortality: Cohort study using the General Practice Research Database. Eur. Heart J..

[71] Schmitt J., Ford D.E. (2010). Psoriasis is independently associated with psychiatric morbidity and adverse cardiovascular risk factors, but not with cardiovascular events in a population-based sample. J. Eur. Acad. Dermatol. Venereol..

[72] Wakkee M., Herings R.M., Nijsten T. (2010). Psoriasis May Not be an Independent Risk Factor for Acute Ischemic Heart Disease Hospitalizations: Results of a Large Population-Based Dutch Cohort. J. Investig. Dermatol..

[73] Brauchli Y.B., Jick S.S., Miret M., Meier C.R. (2009). Psoriasis and risk of incident myocardial infarction, stroke or transient ischaemic attack: An inception cohort study with a nested case—Control analysis. Br. J. Dermatol..

[74] Prodanovich S., Kirsner R.S., Kravetz J.D., Ma F., Martínez L., Federman D.G. (2009). Association of Psoriasis with Coronary Artery, Cerebrovascular, and Peripheral Vascular Diseases and Mortality. Arch. Dermatol..

[75] Xiao J., Chen L.-H., Tu Y.-T., Deng X.-H., Tao J. (2009). Prevalence of myocardial infarction in patients with psoriasis in central China. J. Eur. Acad. Dermatol. Venereol..

[76] Kaye J., Li L., Jick S. (2008). Incidence of risk factors for myocardial infarction and other vascular diseases in patients with psoriasis. Br. J. Dermatol..

[77] Liu L., Cui S., Liu M., Huo X., Zhang G., Wang N. (2022). Psoriasis Increased the Risk of Adverse Cardiovascular Outcomes: A New Systematic Review and Meta-Analysis of Cohort Study. Front. Cardiovasc. Med..

[78] Samarasekera E.J., Neilson J.M., Warren R.B., Parnham J., Smith C.H. (2013). Incidence of Cardiovascular Disease in Individuals with Psoriasis: A Systematic Review and Meta-Analysis. J. Investig. Dermatol..

[79] Armstrong E.J., Harskamp C.T., Armstrong A.W. (2013). Psoriasis and Major Adverse Cardiovascular Events: A Systematic Review and Meta-Analysis of Observational Studies. J. Am. Heart Assoc..

[80] Arnett D.K., Blumenthal R.S., Albert M.A., Buroker A.B., Goldberger Z.D., Hahn E.J., Himmelfarb C.D., Khera A., Lloyd-Jones D., McEvoy J.W. (2019). 2019 ACC/AHA guideline on the primary prevention of cardiovascular disease: A report of the American College of Cardiology/American Heart Association Task Force on Clinical Practice Guidelines. Circulation.

[81] Bradley C.K., Wang T.Y., Li S., Robinson J.G., Roger V.L., Goldberg A.C., Virani S.S., Louie M.J., Lee L.V., Peterson E.D. (2019). Patient-Reported Reasons for Declining or Discontinuing Statin Therapy: Insights from the PALM Registry. J. Am. Heart Assoc..

[82] Barbieri J.S., Beidas R.S., Gondo G.C., Fishman J., Williams N.J., Armstrong A.W., Ogdie A.R., Mehta N., Gelfand J.M. (2022). Analysis of Specialist and Patient Perspectives on Strategies to Improve Cardiovascular Disease Prevention among Persons with Psoriatic Disease. JAMA Dermatol..

[83] Elmets C.A., Leonardi C.L., Davis D.M., Gelfand J.M., Lichten J., Mehta N.N., Armstrong A.W., Connor C., Cordoro K.M., Elewski B.E. (2019). Joint AAD-NPF guidelines of care for the management and treatment of psoriasis with awareness and attention to comorbidities. J. Am. Acad. Dermatol..

[84] Garshick M.S., Berger J.S. (2022). Psoriasis and Cardiovascular Disease—An Ounce of Prevention Is Worth a Pound of Cure. JAMA Dermatol..

[85] Hansson G.K. (2017). Inflammation and Atherosclerosis: The End of a Controversy. Circulation.

[86] Hansson G.K. (2005). Inflammation, Atherosclerosis, and Coronary Artery Disease. N. Engl. J. Med..

[87] Libby P., Buring J.E., Badimon L., Hansson G.K., Deanfield J., Bittencourt M.S., Tokgözoğlu L., Lewis E.F. (2019). Atherosclerosis. Nat. Rev. Dis. Primers.

[88] Kishikawa H., Shimokama T., Watanabe T. (1993). Localization of T lymphocytes and macrophages expressing IL-1, IL-2 receptor, IL-6 and TNF in human aortic intima. Role of cell-mediated immunity in human atherogenesis. Virchows Arch..

[89] Tipping P.G., Hancock W.W. (1993). Production of tumor necrosis factor and interleukin-1 by macrophages from human atheromatous plaques. Am. J. Pathol..

[90] Fernandez D.M., Rahman A.H., Fernandez N.F., Chudnovskiy A., Amir E.-A.D., Amadori L., Khan N.S., Wong C.K., Shamailova R., Hill C.A. (2019). Single-cell immune landscape of human atherosclerotic plaques. Nat. Med..

[91] Nus M., Mallat Z. (2016). Immune-mediated mechanisms of atherosclerosis and implications for the clinic. Expert Rev. Clin. Immunol..

[92] Libby P., Hansson G.K. (2015). Inflammation and Immunity in Diseases of the Arterial Tree. Circ. Res..

[93] Liuzzo G., Trotta F., Pedicino D. (2013). Interleukin-17 in atherosclerosis and cardiovascular disease: The good, the bad, and the unknown: Players and layers. Eur. Heart J..

[94] Ridker P.M., Danielson E., Fonseca F.A., Genest J., Gotto A.M., Kastelein J.J., Koenig W., Libby P., Lorenzatti A.J., MacFadyen J.G. (2008). Rosuvastatin to prevent vascular events in men and women with elevated C-reactive protein. N. Engl. J. Med..

[95] Garshick M.S., Drenkova K., Barrett T.J., Schlamp F., Fisher E.A., Katz S., Jelic S., Neimann A.L., Scher J.U., Krueger J. (2022). A Randomized Open-Label Clinical Trial of Lipid-Lowering Therapy in Psoriasis to Reduce Vascular Endothelial Inflammation. J. Investig. Dermatol..

[96] Elnabawi Y.A., Oikonomou E., Dey A.K., Mancio J., Rodante J.A., Aksentijevich M., Choi H., Keel A., Erb-Alvarez J., Teague H.L. (2019). Association of Biologic Therapy with Coronary Inflammation in Patients with Psoriasis as Assessed by Perivascular Fat Attenuation Index. JAMA Cardiol..

[97] Jókai H., Szakonyi J., Kontár O., Marschalkó M., Szalai K., Kárpáti S., Holló P. (2013). Impact of effective tumor necrosis factor-alfa inhibitor treatment on arterial intima-media thickness in psoriasis: Results of a pilot study. J. Am. Acad. Dermatol..

[98] Martinez-Lopez A., Blasco-Morente G., Perez-Lopez I., Tercedor-Sanchez J., Arias-Santiago S. (2018). Studying the effect of systemic and biological drugs on intima-media thickness in patients suffering from moderate and severe psoriasis. J. Eur. Acad. Dermatol. Venereol..

[99] Marovt M., Marko P., Pirnat M., Ekart R. (2020). Effect of biologics targeting interleukin-23/-17 axis on subclinical atherosclerosis: Results of a pilot study. Clin. Exp. Dermatol..

[100] Piros É.Á., Szabó Á., Rencz F., Brodszky V., Szalai K., Galajda N., Szilveszter B., Dósa E., Merkely B., Holló P. (2021). Impact of Interleukin-17 Inhibitor Therapy on Arterial Intima-media Thickness among Severe Psoriatic Patients. Life.

[101] Ahlehoff O., Skov L., Gislason G., Gniadecki R., Iversen L., Bryld L., Lasthein S., Lindhardsen J., Kristensen S., Torp-Pedersen C. (2015). Cardiovascular outcomes and systemic anti-inflammatory drugs in patients with severe psoriasis: 5-year follow-up of a Danish nationwide cohort. J. Eur. Acad. Dermatol. Venereol..

[102] Tan E., Baker C., Foley P. (2013). Weight gain and tumour necrosis factor-alpha inhibitors in patients with psoriasis. Australas. J. Dermatol..

[103] Gisondi P., Cotena C., Tessari G., Girolomoni G. (2008). Anti–tumour necrosis factor-α therapy increases body weight in patients with chronic plaque psoriasis: A retrospective cohort study. J. Eur. Acad. Dermatol. Venereol..

[104] Al-Mutairi N., Shabaan D. (2016). Effects of tumor necrosis factor α inhibitors extend beyond psoriasis: Insulin sensitivity in psoriasis patients with type 2 diabetes mellitus. Cutis.

[105] Ursini F., Leporini C., Bene F., D’Angelo S., Mauro D., Russo E., De Sarro G., Olivieri I., Pitzalis C., Lewis M. (2017). Anti-TNF-alpha agents and endothelial function in rheumatoid arthritis: A systematic review and meta-analysis. Sci. Rep..

[106] Yang Z.-S., Lin N.-N., Li L., Li Y. (2016). The Effect of TNF Inhibitors on Cardiovascular Events in Psoriasis and Psoriatic Arthritis: An Updated Meta-Analysis. Clin. Rev. Allergy Immunol..

[107] Shaaban D., Al-Mutairi N. (2018). The effect of tumor necrosis factor inhibitor therapy on the incidence of myocardial infarction in patients with psoriasis: A retrospective study. J. Dermatol. Treat..

[108] Erbel C., Dengler T.J., Wangler S., Lasitschka F., Bea F., Wambsganss N., Hakimi M., Böckler D., Katus H.A., Gleissner C.A. (2011). Expression of IL-17A in human atherosclerotic lesions is associated with increased inflammation and plaque vulnerability. Basic Res. Cardiol..

[109] Fatkhullina A.R., Peshkova I.O., Dzutsev A., Aghayev T., McCulloch J.A., Thovarai V., Badger J.H., Vats R., Sundd P., Tang H.-Y. (2018). An Interleukin-23-Interleukin-22 Axis Regulates Intestinal Microbial Homeostasis to Protect from Diet-Induced Atherosclerosis. Immunity.

[110] Engelbertsen D., Depuydt M.A.C., Verwilligen R.A.F., Rattik S., Levinsohn E., Edsfeldt A., Kuperwaser F., Jarolim P., Lichtman A.H. (2018). IL-23R Deficiency Does Not Impact Atherosclerotic Plaque Development in Mice. J. Am. Heart Assoc..

[111] Gaffen S.L. (2009). Structure and signalling in the IL-17 receptor family. Nat. Rev. Immunol..

[112] Reich K., Pinter A., Lacour J., Ferrandiz C., Micali G., French L., Lomaga M., Dutronc Y., Henneges C., Wilhelm S. (2017). Comparison of ixekizumab with ustekinumab in moderate-to-severe psoriasis: 24-week results from IXORA-S, a phase III study. Br. J. Dermatol..

[113] Lebwohl M., Strober B., Menter A., Gordon K., Weglowska J., Puig L., Papp K., Spelman L., Toth D., Kerdel F. (2015). Phase 3 Studies Comparing Brodalumab with Ustekinumab in Psoriasis. N. Engl. J. Med..

